# Rising incidence of acute total hip arthroplasty for primary and adjunctive treatment of acetabular fracture in older and middle-aged adults

**DOI:** 10.1007/s00590-023-03653-4

**Published:** 2023-07-22

**Authors:** Joseph T. Patterson, Julian Wier, Pranit Kumaran, Andrew Adamczyk

**Affiliations:** 1 Department of Orthopaedic Surgery, Keck School of Medicine of the University of Southern California, 1520 San Pablo Street, Suite 2000, Los Angeles, CA 90033-5322 USA; 2 Department of Orthopaedic Surgery, University of Arizona College of Medicine Phoenix, Phoenix, AZ USA

**Keywords:** Acetabular fracture, Total hip arthroplasty, Open reduction internal fixation, Combined hip procedure, Adjunctive, Middle-aged, Older adults, Medicare, United States, Nationwide Inpatient Sample

## Abstract

**Background:**

Acute total hip arthroplasty (THA) may be an alternative or an adjuvant to internal fixation for surgical treatment of acetabular fractures. We investigate recent trends in the operative management of acetabular fractures. We hypothesize that the incidence of acute THA for acetabular fractures has increased over time.

**Methods:**

4569 middle-aged (45–64 years) and older adults (≥ 65 years) who received acute operative management of an acetabular fracture within 3 weeks of admission between 2010 and 2020 were identified from the United States Nationwide Inpatient Sample database. Treatment was classified as open reduction internal fixation (ORIF), THA, or combined ORIF and THA (ORIF + THA). Patients were stratified by age ≥ 65 years old. Associations between demographic factors and the incidence of each procedure over the study period were modeled using linear regression.

**Results:**

The relative incidence of treatments was 80.9% ORIF, 12.1% THA, and 7.0% ORIF + THA. Among patients aged 45–64 years old, THA increased 4.8% [R^2^ = 0.62; β_1_ = 0.6% (95% Confidence Interval (CI) 0.2–0.9%)] and ORIF + THA increased 2.6% [R^2^ = 0.73; β_1_ = 0.3% (95% CI 0.2–0.4%)], while the use of ORIF decreased 7.4% [R^2^ = 0.75; β_1_ = −0.9% (95% CI −1.2 to −0.5%)]. Among patients ≥ 65 years old, THA increased 16.5% [R^2^ = 0.87; β_1_ = 1.7% (95% CI 1.2–2.2%)] and ORIF + THA increased 5.0% [R^2^ = 0.38, β_1_ = 0.6% (95% CI 0.0–1.3%)], while ORIF decreased 21.5% [R^2^ = 0.75; β_1_ = −2.4% (95% CI −3.45 to −1.3%)].

**Conclusion:**

The treatment of acetabular fractures with acute THA has increased in the last decade, particularly among older adults.

**Supplementary Information:**

The online version contains supplementary material available at 10.1007/s00590-023-03653-4.

## Introduction

Acetabular fractures in older patients are occurring more frequently and are associated with high rates of mortality and non-fatal complications [1–3]. These older patients often have pre-existing hip osteoarthritis, osteoporosis, and fracture characteristics predictive of post-traumatic arthritis [4, 5]. Surgical treatment options for older patients with acetabular fracture include open reduction internal fixation (ORIF), total hip arthroplasty (THA), or the combined used of ORIF and THA (ORIF + THA) [6, 7].

While ORIF has been the historical standard of care [6, 8, 9], recent evidence suggests that acute THA may provide better functional outcomes with fewer reoperations in older patients with acetabular fracture [10–17]. It is not known whether surgeons who treat acetabular fractures have changed their practice to perform arthroplasty more frequently. Our purpose is to investigate if the acute use of THA for acetabular fractures in middle-aged and older adults—patients for whom an elective total hip arthroplasty would represent a reasonable intervention for osteoarthritis—has increased over time. We hypothesize that the relative incidence of THA and ORIF + THA have increased in the last decade compared with ORIF as a standard of care. We further characterize facility, patient, and fracture characteristics that may be associated with the choice to manage acetabular fracture with THA as a primary treatment or with adjunctive ORIF.

## Materials and methods

### Data source

Adults ≥ 45 years of age treated for acetabular fracture within 3 weeks of admission between 2010 through 2020 were retrospectively identified from the Nationwide Inpatient Sample (NIS). The NIS is curated by the Healthcare Cost and Utilization Project (HCUP) and includes data on over 7 million hospital discharges per year [18, 19]. In 2012 the NIS included a 20% stratified sample of inpatient admissions across the United States, while prior to 2012 the NIS sampled 20% of discharges from participating hospitals. This methodologic change was intended to improve the precision of national estimates by reducing sampling error [20]. Deidentified publicly available data were used for this investigation, thus this study was exempt from Institutional Review Board review.

### Study population

International Classification of Diseases Ninth Edition Clinical Modification (ICD-9-CM) and Tenth Edition Clinical Modification (ICD-10-CM) codes were used to identify patients with acetabular fractures (Supplementary Table S1). Operative treatments including open reduction internal fixation (ORIF), total hip arthroplasty (THA), and combined ORIF and THA (ORIF + THA) were identified using ICD-9 and ICD-10 procedure codes (PCS; Supplementary Table S2). Patients < 45 years old and those without recorded operative procedure or procedures > 3 weeks after admission were excluded to capture patients with acute injuries and those who are most likely to benefit from and be treated by acute THA [21–23].

A weighted sample estimate representing 22,795 adults met inclusion criteria (Supplementary Fig. S1). The relative incidence of each treatment was 80.9% ORIF, 12.1% THA, and 7.0% ORIF + THA. Patients receiving each treatment significantly differed by age, sex, insurance type, hospital size, hospital type, and comorbidities overall (Tables 1, 2, Supplemental Table S3-S4).

**Table 1:** Patient characteristics, length of stay, and costs of acetabular fracture surgery by procedure (ages ≥ 65 years)

	ORIF N = 5860			THA N = 1844			ORIF + THA N = 1162			*P*-value
	Average	95% CI Lower	95% CI Upper	Average	95% CI Lower	95% CI Upper	Average	95% CI Lower	95% CI Upper	
Age (years)	75.5	75.0	75.9	77.6	76.9	78.3	77.2	76.3	78.0	< 0.001
Elixhauser comorbidity index	2.8	2.7	2.9	3.0	2.8	3.2	3.1	2.9	3.4	< 0.001

	Number	Percent of ORIF (%)	Percent of row (%)	Number	Percent of THA (%)	Percent of row (%)	Number	Percent of ORIF + THA (%)	Percent of row (%)	
Male sex	3768	64.4	79.0	541	29.5	11.3	462	39.9	9.7	< 0.001
*Race*										
Asian or PI	76	1.3	79.2	20	1.1	20.8	0	0.0	0.0	0.04
Black	384	6.6	73.3	75	4.1	14.3	65	5.6	12.4	
Hispanic	219	3.8	66.7	60	3.2	18.2	50	4.3	15.1	
Other	648	11.1	75.0	113	6.2	13.1	103	9.0	11.9	
White	4533	77.4	64.3	1576	85.4	22.3	944	81.1	13.4	
*Insurance type*										
Medicare	4293	73.3	62.6	1625	88.1	23.7	944	81.1	13.8	< 0.001
Medicaid	35	0.6	49.7	5	0.3	7.1	30	2.6	43.2	
Private	1193	20.4	81.1	158	8.6	10.8	119	10.3	8.1	
Self-Pay	89	1.5	69.1	15	0.8	11.6	25	2.1	19.3	
Other	245	4.2	76.7	36	1.9	11.1	39	3.4	12.1	
*Hospital type*										
Rural	203	3.5	52.0	138	7.6	35.3	50	4.3	12.7	< 0.001
Urban, nonteaching	573	9.9	49.3	429	23.2	36.9	160	13.7	13.8	
Urban, teaching	5069	86.4	69.5	1277	69.2	17.5	952	82.0	13.0	
Other	15	0.3	100.0	0	0.0	0.0	0	0.0	0.0	
*Region*										
Northeast	920	15.6	61.2	351	19.2	23.4	233	20.2	15.5	0.002
Midwest	1336	22.9	61.1	468	25.4	21.4	383	33.0	17.5	
South	2460	42.1	70.7	664	35.9	19.1	356	30.5	10.2	
West	1144	19.5	67.5	361	19.5	21.3	190	16.3	11.2	

Average	95% CI Lower	95% CI Upper	Average	95% CI Lower	95% CI Upper	Average	95% CI Lower	95% CI Upper	
*Length of stay (days)*									
9.5	9.1	9.9	6.1	5.6	6.7	8.0	7.4	8.6	< 0.001
*Total cost ($)*									
$ 138,063.30	$ 131,015.00	$ 145,111.60	$ 111,915.40	$ 103,521.60	$ 120,309.10	$ 153,841.50	$ 139,543.00	$ 168,139.90	< 0.001

*ORIF* open reduction internal fixation, *THA* total hip arthroplasty, *CI* confidence interval

**Table 2:** Patient characteristics, length of stay, and costs of acetabular fracture surgery by procedure (ages 45–64 years)

	ORIF N = 12,588			THA N = 906			ORIF + THA N = 435			*P*-value
	Average	95% CI Lower	95% CI Upper	Average	95% CI Lower	95% CI Upper	Average	95% CI Lower	95% CI Upper	
Age (years)	54.6	54.3	54.8	57.6	56.7	58.4	58.9	57.8	60.0	< 0.001
Elixhauser comorbidity index	1.9	1.8	1.9	2.6	2.3	2.9	2.6	2.2	2.9	< 0.001

	Number	Percent of ORIF (%)	Percent of row (%)	Number	Percent of THA (%)	Percent of row (%)	Number	Percent of ORIF + THA (%)	Percent of row (%)	
Male sex	9136	72.6	94.0	369	40.7	3.8	210	48.3	2.2	< 0.001
*Race*										
Asian or PI	199	1.6	95.2	5	0.5	2.4	5	1.1	2.4	0.17
Black	1522	12.1	93.2	85	9.3	5.2	25	5.7	1.5	
Hispanic	834	6.6	93.3	45	4.9	5.0	15	3.4	1.7	
Other	1290	10.3	91.5	65	7.1	4.6	56	12.6	3.9	
White	8743	69.5	89.4	706	78.0	7.2	334	77.0	3.4	
*Insurance type*										
Medicare	1203	9.6	76.2	280	30.8	17.7	95	21.8	6.0	< 0.001
Medicaid	1649	13.1	89.5	144	15.9	7.8	49	11.5	2.7	
Private	6751	53.6	91.1	401	44.5	5.4	255	58.6	3.4	
Self-Pay	1195	9.5	96.8	35	3.8	2.8	5	1.1	0.4	
Other	1725	13.7	95.8	46	4.9	2.6	30	6.9	1.7	
*Hospital type*										
Rural	397	3.2	79.4	79	8.8	15.8	24	5.7	4.8	< 0.001
Urban, nonteaching	917	7.4	80.3	180	19.8	15.7	45	10.3	3.9	
Urban, teaching	11,192	88.8	91.8	643	70.9	5.3	360	82.8	3.0	
Other	82	0.6	88.3	5	0.5	5.6	6	1.1	6.1	
*Region*										
Northeast	1836	14.6	91.2	98	11.0	4.9	80	18.4	4.0	0.008
Midwest	3005	23.9	88.1	245	26.9	7.2	160	36.8	4.7	
South	5226	41.6	90.9	418	46.2	7.3	104	24.1	1.8	
West	2520	19.9	91.4	145	15.9	5.3	91	20.7	3.3	

	Average	95% CI Lower	95% CI Upper	Average	95% CI Lower	95% CI Upper	Average	95% CI Lower	95% CI Upper	
Length of Stay (days)	9.8	9.4	10.1	6.9	5.4	8.5	8.7	6.0	11.5	< 0.001
Total Cost ($)	$ 146,089.20	$ 140,166.80	$ 152,011.60	$ 113,985.30	$ 97,938.96	$ 130,031.70	$ 175,615.70	$ 136,192.10	$ 215,039.40	< 0.001

*ORIF* open reduction internal fixation, *THA* total hip arthroplasty, *CI* confidence interval

### Study outcomes

The primary outcome was annual change in the incidence of ORIF, THA, and ORIF + THA. Secondary outcomes included hospital length of stay and national charge estimates for cost of hospital stay.

### Statistical methods

Annual procedure incidence was modeled using linear regression on proportional changes in yearly cases. Patients were stratified by age 45–64 years (middle-aged) or ≥ 65 years (older adult). Further subgroup analysis considered the effects of sex and insurance type within age groups on treatment. Chow tests were conducted to determine differences in linear regression coefficients β across groups. Adjusted R^2^ was used to determine goodness-of-fit. Continuous and categorical variables were compared across unweighted treatment groups using one way analysis of variance and chi-squared, respectively. National charge estimates for hospital stay were derived using the NIS discharge weighting scheme which reflects a ratio of national discharges to discharges captured by NIS. Statistical analyses were performed using Stata Version 17.0 (College, Station, TX), reporting 2-sided p values with the level of significance for *p* < 0.050.

## Results

### Treatment by age

For patients aged ≥ 65 years, 5860 (65.8%) underwent ORIF, 1844 (20.8%) underwent THA, and 1162 (13.1%) underwent ORIF + THA. For patients aged 45–64 years, 12,588 (90.4%) underwent ORIF, 906 (6.5%) underwent THA, and 435 (3.1%) underwent ORIF + THA. We observed greater overall use of THA (20.8%) and ORIF + THA (13.1%) for patients ≥ 65 years with acetabular fracture versus greater use of ORIF (90.4%) in the 45–64 group (Table 1). The mean age of the ≥ 65 group was 76.1 years (95% confidence interval (CI) 75.8–76.5 years), with those undergoing ORIF (75.5 years; 95% CI 75.0–75.9 years) being significantly younger than those undergoing THA (77.6 years; 95% CI 77.6–78.3 years) or ORIF + THA (77.2 years; 95% CI 76.3–78.0 years) (*p* < 0.001) (Table 1). The mean age of the 45–64 group was 54.9 years (95% CI 54.7–55.1 years), with significantly younger patients undergoing ORIF (54.6 years; 54.3–54.8 years) than those undergoing THA (57.6 years; 95% CI 56.7–58.4 years) or ORIF + THA (58.9 years; 95% CI 57.8–60.0 years) (Table 2).

### Treatment trends by age

**Fig. 1:**
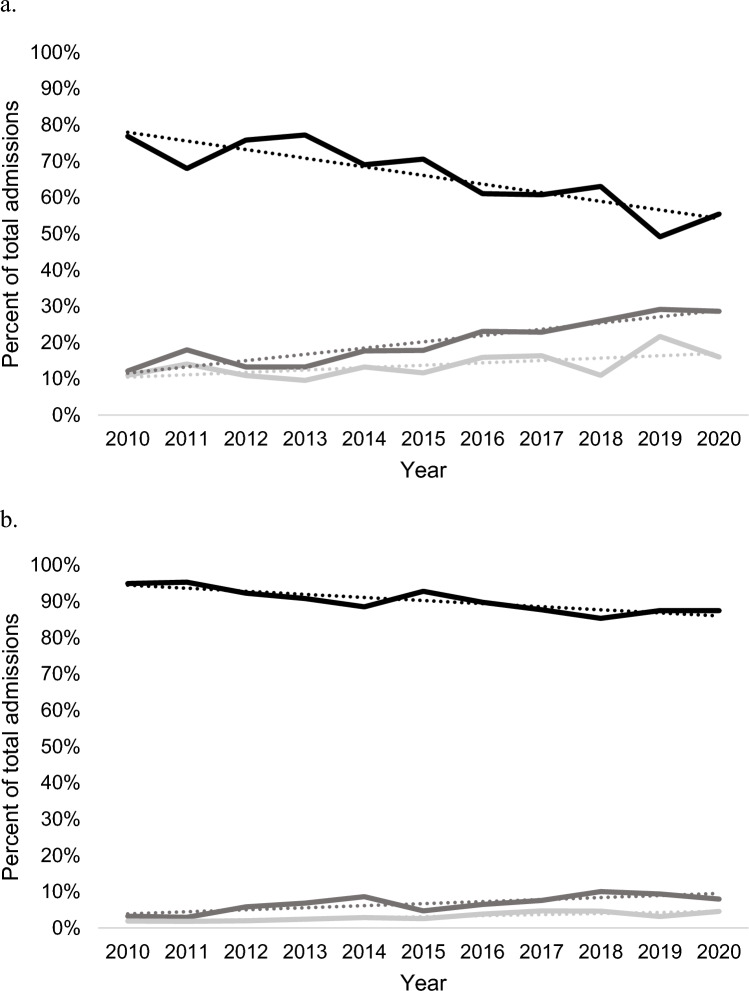
Acetabular treatment trends by open reduction internal fixation (black), total hip arthroplasty (dark grey), and open reduction internal fixation + total hip arthroplasty (light grey) in the NIS database from 2010 to 2020. **A** In ages ≥ 65.  **B** In ages 45–64

We observed an increase in the use of THA for acetabular fracture over the study period in middle-aged and older adults. From 2010 to 2020, the use of ORIF for patients ≥ 65 years decreased 21.5% [R^2^ = 0.75; β_1_ = −2.4% per year (95% CI −3.4 to −1.3%)], while THA increased 16.5% [R^2^ = 0.87; β_1_ = 1.7% per year (95% CI 1.2–2.2%)] and ORIF + THA increased 5.0% [R^2^ = 0.38, β_1_ = 0.6% per year (95% CI 0.0–1.3%)] (Fig. 1a). Among patients aged 45–64 years, the use of ORIF decreased 7.4% [R^2^ = 0.75; β_1_ = −0.9% per year (95% CI −1.2 to −0.5%)], while THA increased 4.8% [R^2^ = 0.62; β_1_ = 0.6% per year (95% CI 0.2–0.9%)] and ORIF + THA increased 2.6% [R^2^ = 0.73; β_1_ = 0.3% per year (95% CI 0.2–0.4%)], (Fig. 1b). Annual rates of change in practice were significantly different between age groups for ORIF (*p* < 0.001) and THA (*p* < 0.001), but not for ORIF + THA (*p* > 0.05).

### Treatment trends by sex

**Fig. 2:**
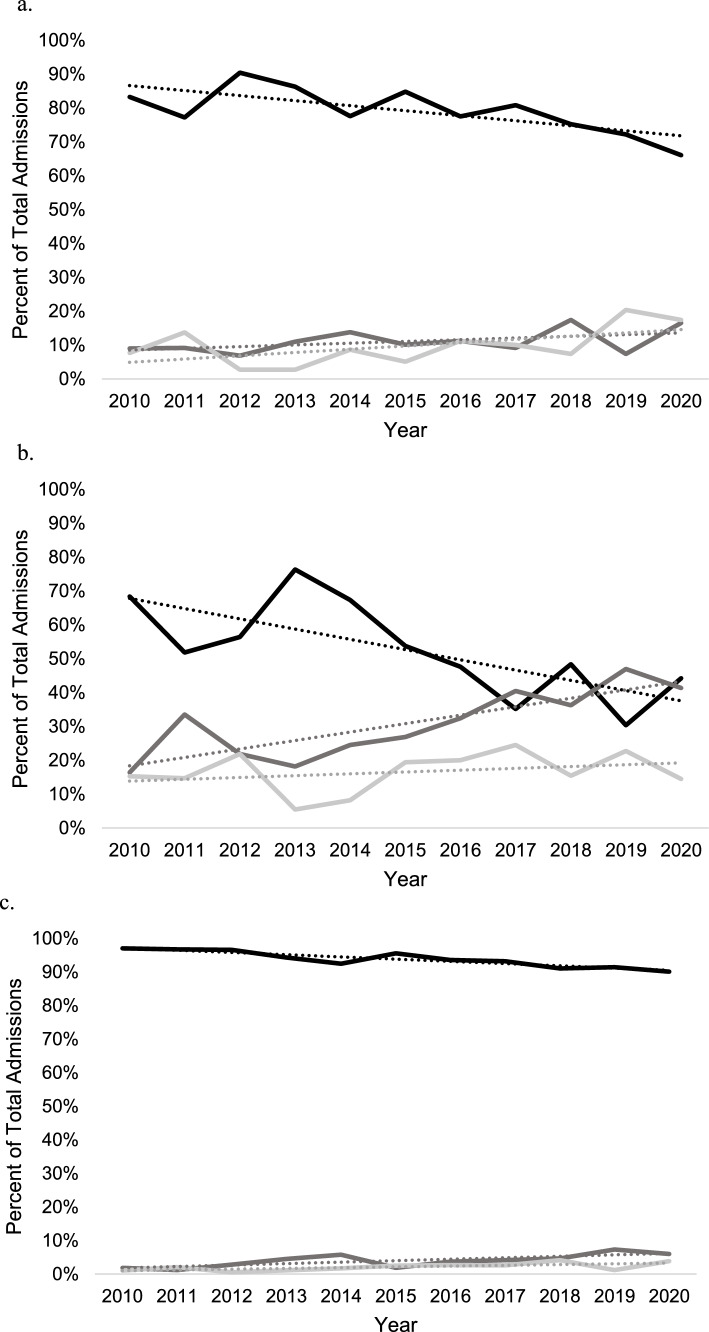

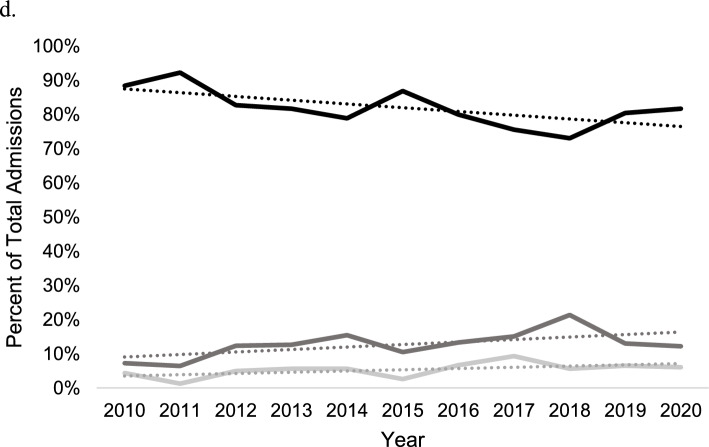
Acetabular treatment trends by open reduction internal fixation (black), total hip arthroplasty (dark grey), and open reduction internal fixation + total hip arthroplasty (light grey) in the NIS database from 2010 to 2020. **A** males in ages ≥ 65. **B** Females in ages ≥ 65.  **C** Males in ages 45–64.** d** Females in ages 45–64

Patient sex was associated with changes in practice over time. Acute THA for acetabular fracture significantly increased over time among women ≥ 65 years [R^2^ = 0.68; β_1_ = 2.5% per year (95% CI 1.2–3.8%)]. Treatment by ORIF and ORIF + THA did not change over time among women ≥ 65 years. No temporal change of practice was observed among men [R^2^ = 0.24; β_1_ = 0.5% per year (95% CI −0.2 to 1.2%); *p* = 0.002; Fig. 2a, b]. No differences in treatment by sex were observed over time among patients aged 45–64 years (*p* > 0.05; Fig. 2c, d).

### Treatment trends by insurance type

**Fig. 3:**
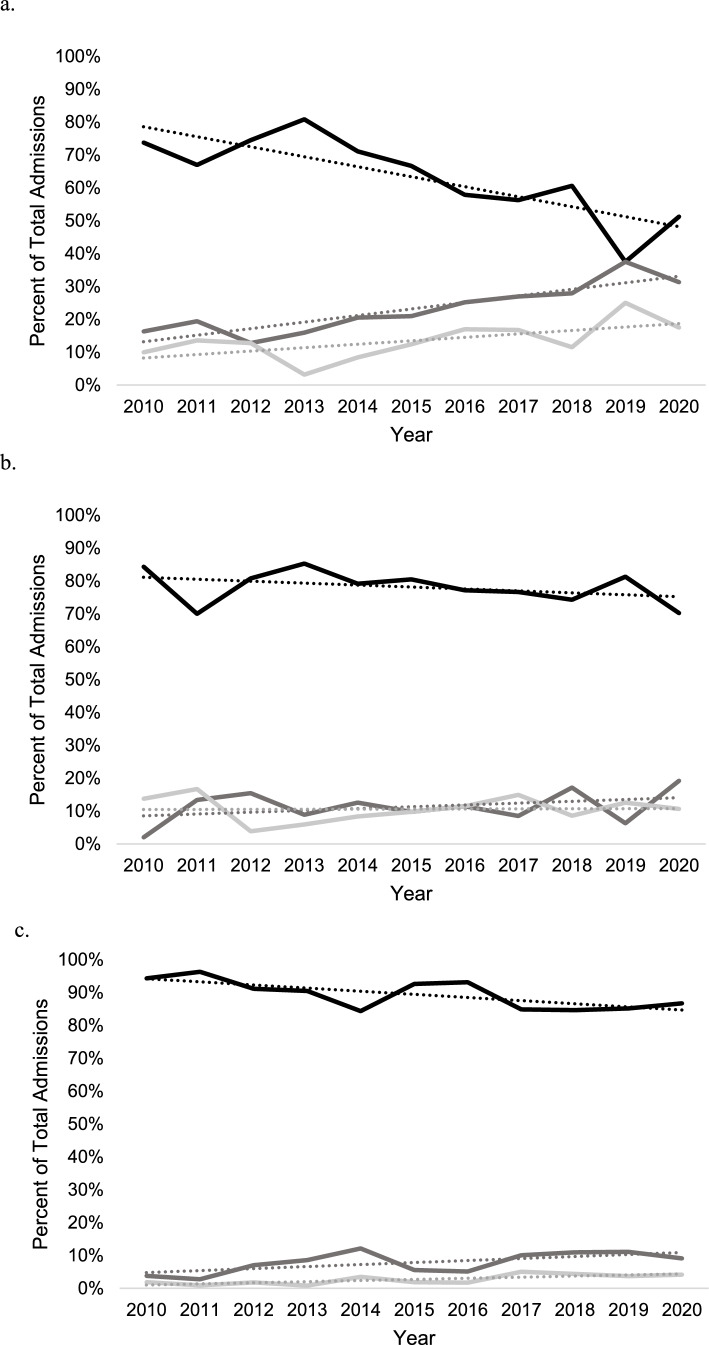

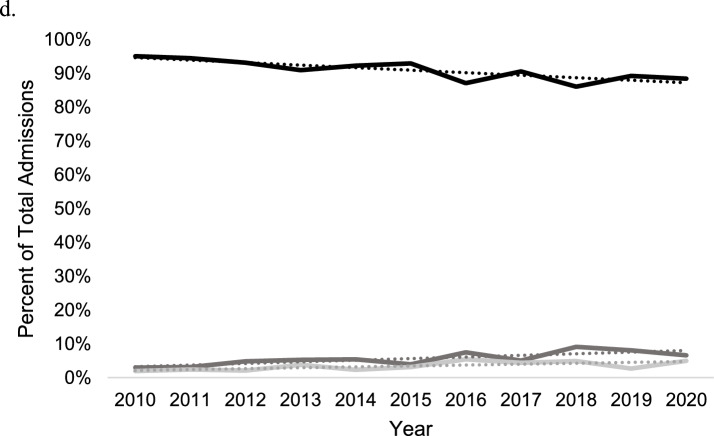
Acetabular treatment trends by open reduction internal fixation (black), total hip arthroplasty (dark grey), and open reduction internal fixation + total hip arthroplasty (light grey) in the NIS database from 2010 to 2020. **A** Medicare insured in ages ≥ 65. **B** Non-Medicare insured in ages ≥ 65. **C** Privately insured in ages 45–64.** d** Non-privately insured in ages 45–64

Medicare insurance status was associated with the greatest growth in use of THA in acetabular fracture among patients aged ≥ 65 years [R^2^ = 0.80; β_1_ = 2.0% per year (95% CI = 1.2–2.7%) vs R^2^ = 0.14; β_1_ = 0.6% per year (95% CI = −0.5 to 1.6%); *p* = 0.006]. Additionally, the relative use of ORIF declined more rapidly over time in the Medicare insured patients (R^2^ = 0.67; β_1_ = −3.0% per year (95% CI = −4.7 to −1.4%)) when compared to non-Medicare insured patients [R^2^ = 0.15; β_1_ = −0.6% per year (95% CI = −1.7–0.5%); *p* = 0.03]. No differences in longitudinal ORIF + THA use rates were observed between insurance groups (*p* > 0.05; Fig. 3a, b). Insurance type was not significantly associated with treatment in patients aged 45–64 years (*p* > 0.05; Fig. 3c, d).

### Treatment trends by hospital type

Hospital type was associated with treatment but not significantly associated with changes in practice over time. The incidence of THA and THA + ORIF increased over time in rural, urban non-teaching, and urban teaching hospitals over time, but the rates of practice change were not significantly different across hospital types among patients aged ≥ 65 years (Supplementary Fig. S2a–S3c) or patients 45–64 years old (Supplementary Fig. S2d–S3f).

### Trends in length of stay by treatment

**Fig. 4:**
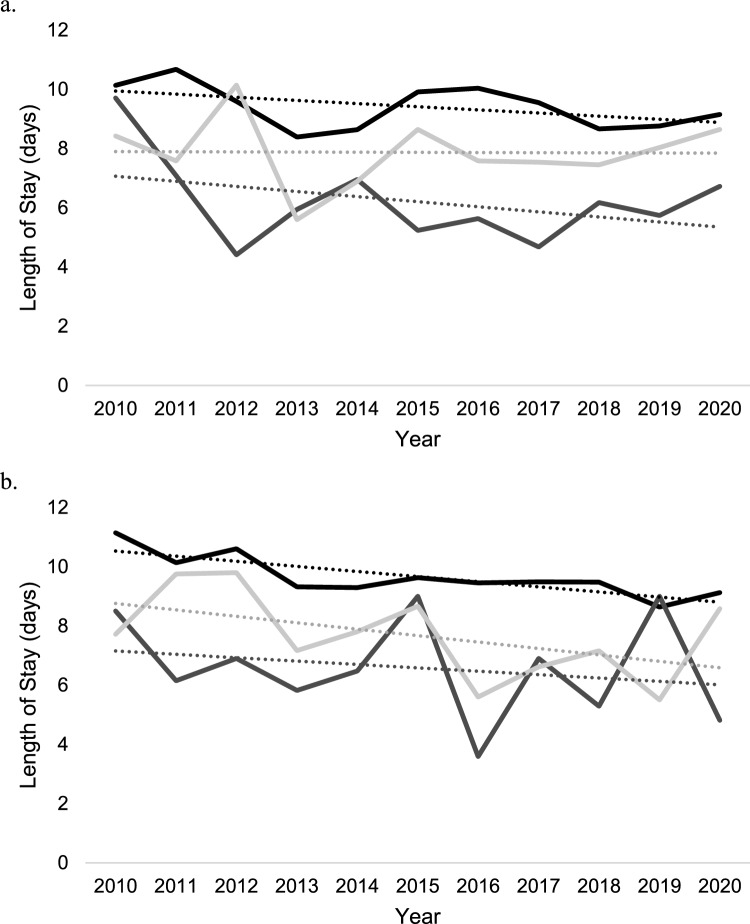
Total hospital length of stay trends in days by open reduction internal fixation (black), total hip arthroplasty (dark grey), and open reduction internal fixation + total hip arthroplasty (light grey) in the NIS database from 2010 to 2020.** a** In ages ≥ 65.** b** In ages 45–64

Length of stay was significantly shorter among patients who received THA [6.4 days (95% CI 5.8–7.0 days)] versus patients who received ORIF [9.7 days (95% CI 9.4–9.9 days); *p* < 0.001) or ORIF + THA (8.2 days (95% CI 7.3–9.0 days); *p* = 0.004]. Length of stay did not significantly vary over time by procedure among patients ≥ 65 years old with acetabular fracture (Fig. 4a). In patients aged 45–64 years, the overall hospital length of stay decreased over time for ORIF [R^2^ = 0.65; β_1_ = −0.17 days per year (95% CI −0.27 to −0.01 days)] but remained unchanged for both THA [R^2^ = 0.05; β_1_ = −0.11 days per year (95% CI = −0.5, to 0.27 days)] and ORIF + THA [R^2^ = 0.24; β_1_ = −0.22 days per year (95% CI −0.5 to 0.1 days)] (Fig. 4b).

### Trends in estimated charges by treatment

**Fig. 5:**
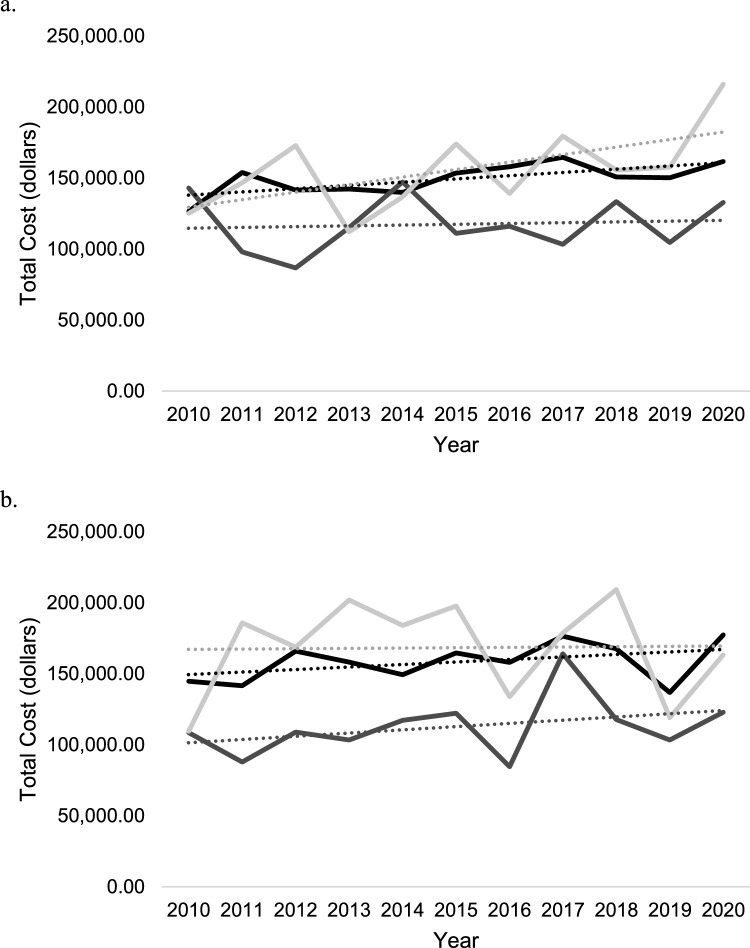
Inflation adjusted total patient cost trends in dollars by open reduction internal fixation (black), total hip arthroplasty (dark grey), and open reduction internal fixation + total hip arthroplasty (light grey) in the NIS database from 2010 to 2020.** a** In ages ≥ 65.** b** In ages 45–64

National estimated hospital charges were significantly lower in patients who received THA [$112,591.40 (95% CI $104,912.40–$120,270.40)] compared to patients who received ORIF [$143,542.80 (95% CI $138,922.50–$148,163.10)] (*p* < 0.001) and ORIF + THA [$159,730.10 (95% CI $144,901.70–$174,558.60)] (*p* < 0.001). In patients ≥ 65 years old, the cost of both ORIF [R^2^ = 0.47; β_1_ = $2285.59 per year (95% CI $456.96–$4114.22)] and ORIF + THA [R^2^ = 0.37; β_1_ = $5308.89 per year (95% CI $75.24–$10,542.53)] increased significantly per year, while the cost of THA [R^2^ = 0.01; β_1_ = $550.01 per year (95% CI −$3844.19–$4944.22)] did not change (Fig. 5a). In patients 45–64 years old, no differences in cost of ORIF [R^2^ = 0.19; β_1_ = $1762.39 per year (95% CI −$1062.08–$4586.87)], THA [R^2^ = 0.13; β_1_ = $2,272.71 per year (95% CI −$2234.15–$6779.56)], or ORIF + THA [R^2^ = 0.00; β_1_ = $245.90 per year (95% CI −$7443.65–$7935.46)] were observed over time (Fig. 5b).

## Discussion

We observed substantial practice changes in the surgical management of middle-aged and older US adults with acetabular fracture between 2010 and 2020 in the Nationwide Inpatient Sample. Among older adults aged ≥ 65 years, the incidence of THA and ORIF + THA increased 16.5% and 5.0%, respectively, while the use of ORIF decreased by 21.5%. Practice changes were less substantial among middle-aged adults aged 45–64 years: the incidence of THA and ORIF + THA increased 4.8% and 2.6%, respectively, while the use of ORIF decreased 7.4%. The practice of acute THA for acetabular fracture grew 1.6% annually among older adults and 0.6% annually among middle-aged adults. To our knowledge, these novel observations reflect progressive adoption of arthroplasty for acetabular fractures in middle-aged and older adults.

Acetabular fractures in older adults incur substantial morbidity, disability, and healthcare costs [2, 3]. The adoption of acute THA as either a primary treatment or combined with adjunctive ORIF may reflect a response by surgeons to contemporary evidence for benefits to arthroplasty over ORIF alone in older patients. In a recent systematic review on the outcomes following acetabular fracture in 3928 patients aged 55–99 years, McCormick et al. found ORIF was associated with 2.2 greater odds of non-fatal complications versus THA and a pooled 15% rate of conversion to THA after ORIF at a mean of 29.4 months [12]. Although early reports of ORIF + THA have reported concerning rates of heterotopic ossification, dislocation, and other mechanical failures, single center series suggest improved survival with low rates of reoperation [7, 24]. Manson et al. prospectively randomized 47 patients over age 60 to either ORIF or ORIF + THA and reported a 28% absolute risk reduction in overall reoperations and 12% greater Harris Hip score with no dislocations in either group within 2 years [22]. Borg et al. found improved three-year hip survival in patients undergoing ORIF + THA when compared to ORIF alone [13]. Lin et al. noted equivalent functional outcomes to patients undergoing primary THA for osteoarthritis with a 15% complication rate [17]. However, Kelly et al. identified a 27% surgical complication rate, 13% medical complication rate, and 18% all cause revision risk in a recent review of insurance claims data [25]. These studies provide compelling, if tempered, support for the use of arthroplasty to treat acetabular fractures with a reasonable probability of a favorable outcome in older patients [24].

ORIF remains widely regarded as the standard of care for younger patients with displaced or minimally-displaced but unstable acetabular fractures. We did not observe a clinically relevant change—a minimal 0.9% decline—in the incidence of ORIF for acetabular fracture in middle-aged patients aged 45–64 years. Younger and middle-aged adults experience excellent hip survival and functional outcomes when the congruity and stability of the hip joint are restored [5, 6]. However, specific fracture patterns and patient factors may increase the risk of subsequent posttraumatic arthritis requiring conversion to THA [8, 26, 27]. It is possible that specific fracture and patient characteristics, evolution in implant materials and bearing surfaces, and surgeon confidence in implant survivorship may have driven the small increases in the use of THA (4.8%) and ORIF + THA (2.6%) in middle-aged adults.

We observed differences in the treatment of acetabular fractures by patient sex among older adults: women over age 65 were more likely to receive a THA for acetabular fracture than men, with the incidence of THA for acetabular fracture increasing 2.5% annual in women and 0.5% per year in men. Disparities in treatment by sex may be related to sex-based differences in underlying risk factors for poor outcome with ORIF – chiefly, perhaps, osteoporosis. Most acetabular fractures in the elderly are associated with osteopenic or osteoporotic bone and consequently demonstrate more complex fracture patterns with impaction, comminution, and higher rates of failure when treated with ORIF [5, 26]. Women are more likely to develop osteoporosis than men with advancing age, but the burden of metabolic bone disease among older men and the implications for the outcomes of acetabular ORIF should not be disregarded [28, 29]. The sex-based differences in treatment we observed may also reflect survivorship bias as more women survive to old age and sustain acetabular fractures than men. We did not observe longitudinal trends in treatment by race.

Medicare primary insurance was also associated with a more rapid adoption of THA and concurrent decline in use of ORIF compared to patients without non-Medicare primary insurance. Medicare reimbursement for hip fracture declined 30% between 2000 and 2020 [30]. Medicare reimbursement of acetabular fracture ORIF under DRG 536 is also equivalent to pelvic fracture closed reduction internal fixation, despite major differences in perioperative resources, risk, blood loss, surgical time, and technical difficulty of open acetabular fracture surgery versus minimally invasive percutaneous pelvic ring screw fixation [31]. Unlike femoral neck and head fractures, THA for acetabular fracture is not eligible for bundled payment programs which may protect reimbursement for this use of THA [32]. Our data also demonstrate longitudinally stable costs of care for patients undergoing THA, while ORIF and ORIF + THA related costs rose significantly over the same period. These differential costs are important considerations and are not fully explained by inflation [33]. The combined economic incentives favoring THA may partially account for the practice change observed in patients with acetabular fracture and Medicare primary insurance.

This study has several limitations. The retrospective review of a hospital discharge database is subject to documentation and classification bias. The risk of misclassification may be potentiated by the change from ICD-9 to ICD-10 coding schemes in 2015. We attempted to address this through the careful exclusion of concomitant injuries which may have confounded our analysis. Furthermore, while the NIS database is a validated method to determine national estimates on procedural trends, it is limited to a 20% sample of the actual annual volume of inpatient care. Our observations reflect weighted national estimates rather than observations [34]. This study did not account for injury mechanism, injury severity, or comorbid conditions, which may also influence treatment. These trends may not be generalizable to practice outside of the United States.

In conclusion, THA is increasingly being used as a primary or adjunctive treatment acetabular fracture in middle aged and older US adults, while the incidence of ORIF has declined. Female sex and Medicare insurance were significantly associated with more rapid adoption of THA as a treatment for acetabular fracture in patients ≥ 65 years old. The downstream implications of these evolving treatment trends unknown and warrant further investigation, particularly with regard to physical function, hip survival, health status, independence, and costs of care.

## Supplementary Information

Below is the link to the electronic supplementary material.

Supplementary file 1 (DOCX 31 kb)

STROBE diagram of cohort selection. Given non-specificity of ICD-9-PCS coding for acetabular fractures, patients with concomitant fractures* (clavicular, patellar, pelvic, proximal femoral, sacral, and scapular) were excluded from analysis. (DOCX 25 kb)

Acetabular treatment trends by open reduction internal fixation (black), total hip arthroplasty (dark grey), and open reduction internal fixation + total hip arthroplasty (light grey) in the NIS database from 2010 to 2020 by admitting hospital type. a. rural hospital admissions in ages ≥65 b. urban non-teaching hospital admissions in ages ≥65 c. urban teaching hospital admissions in ages ≥65 d. rural hospital admissions in ages 45–64 e. urban non-teaching hospital admissions in ages 45–64 f. urban teaching hospital admissions in ages 45–64 (DOCX 29 kb)

## Data Availability

Data analyzed in the present study are available online through the National Inpatient Sample Program at https://hcup-us.ahrq.gov/nisoverview.jsp.
