# Supplementary material for: Rising incidence of acute total hip arthroplasty for primary and adjunctive treatment of acetabular fracture in older and middle-aged adults
Source: Eur J Orthop Surg Traumatol. 2023 Jul 22;34(7):3509–21. doi: 10.1007/s00590-023-03653-4 (PMC11490425; doi:10.1007/s00590-023-03653-4)
Supplement: Supplementary file 1 — (DOCX 31 kb) [file 590_2023_3653_MOESM1_ESM.docx]

Supplementary Table S1. International Statistical Classification of Diseases and Related Health Problems 9^th^ and 10^th^ edition clinical modification (CM) codes

|  | ICD-9-CM codes | ICD-10-CM codes |
| --- | --- | --- |
| Acetabular fracture | 8080, 8081 | S32.4 |
| Clavicular fracture | 810 | S42.0 |
| Patellar fracture | 822 | S82.0 |
| Pelvic ring injury | 8082, 8083, 8084, 8085 | S32.3, S32.5, S32.6, S32.8, S32.9 |
| Proximal femoral fracture | 820 | S72.0, S72.1 |
| Sacral fracture | 8056, 8057, 8066, 8067 | S32.1 |
| Scapular fracture | 811 | S42.1 |

Supplementary Table S2. International Statistical Classification of Diseases and Related Health Problems 9^th^ and 10^th^ edition procedure coding system (PCS) codes

|  | ICD-9-PCS codes | ICD-10-PCS codes |
| --- | --- | --- |
| Acetabular ORIF | N/A | 0QS404Z, 0QS504Z, 0QH404Z, 0QH504Z |
| Total hip arthroplasty | 81.51 | 0SR90, 0SRB0 |
| ORIF of other specified bone or repair of hip not elsewhere classified | 79.39, 81.40 | N/A |

ORIF: open reduction internal fixation.

Supplementary Table S3. Elixhauser comorbidity index data (ages ≥65)

|  | **ORIF**  **N=5,860** | | **THA**  **N=1,844** | | **ORIF+THA**  **N=1,162** | | **P-value** |
| --- | --- | --- | --- | --- | --- | --- | --- |
|  | **N** | **%** | **N** | **%** | **N** | **%** |  |
| Alcohol Abuse | 269 | 4.6% | 50 | 2.7% | 50 | 4.3% | 0.002 |
| Arrhythmia | 70 | 1.2% | 61 | 3.3% | 39 | 3.4% | <0.001 |
| Blood Loss Anemia | 61 | 1.0% | 55 | 3.0% | 30 | 2.5% | <0.001 |
| Cerebrovascular Accident | 400 | 6.8% | 116 | 6.3% | 105 | 9.0% | 0.011 |
| Chronic Peptic Ulcer Disease | 10 | 0.2% | 21 | 1.1% | 15 | 1.3% | <0.001 |
| Chronic Pulmonary Disease | 513 | 8.8% | 94 | 5.1% | 73 | 6.3% | <0.001 |
| Coagulopathy | 589 | 10.1% | 95 | 5.2% | 130 | 11.2% | <0.001 |
| Congestive Heart Failure | 623 | 10.6% | 245 | 13.3% | 214 | 18.4% | <0.001 |
| Deficiency Anemia | 578 | 9.9% | 198 | 10.7% | 55 | 4.7% | <0.001 |
| Depression | 576 | 9.8% | 200 | 10.8% | 125 | 10.8% | <0.001 |
| Diabetes, complicated | 509 | 8.7% | 145 | 7.9% | 90 | 7.8% | 0.375 |
| Diabetes, uncomplicated | 1246 | 21.3% | 214 | 11.6% | 163 | 14.0% | <0.001 |
| Drug Abuse | 80 | 1.4% | 35 | 1.9% | 20 | 1.7% | 0.222 |
| Fluid and Electrolyte Disorders | 1914 | 32.7% | 473 | 25.6% | 418 | 35.9% | <0.001 |
| Hemiplegia/Paraplegia | 75 | 1.3% | 15 | 0.8% | 19 | 1.7% | 0.115 |
| HIV/AIDS | 0 | 0.0% | 0 | 0.0% | 0 | 0.0% | N/A |
| Hypertension, complicated | 813 | 13.9% | 358 | 19.4% | 234 | 20.2% | <0.001 |
| Hypertension, uncomplicated | 3185 | 54.3% | 1046 | 56.8% | 591 | 50.9% | <0.001 |
| Hypothyroidism | 769 | 13.1% | 369 | 20.0% | 269 | 23.2% | <0.001 |
| Liver Disease | 114 | 2.0% | 40 | 2.2% | 30 | 2.6% | 0.362 |
| Lymphoma | 34 | 0.6% | 35 | 1.9% | 5 | 0.4% | <0.001 |
| Metastatic Cancer | 34 | 0.6% | 25 | 1.3% | 1 | 0.1% | <0.001 |
| Myocardial Infarction | 413 | 7.0% | 69 | 3.8% | 59 | 5.1% | <0.001 |
| Obesity | 650 | 11.1% | 244 | 13.2% | 119 | 10.3% | 0.0166 |
| Osteoporosis | 691 | 11.8% | 329 | 17.9% | 176 | 15.1% | <0.001 |
| Other neurological disorders | 607 | 10.4% | 205 | 11.1% | 149 | 12.8% | 0.0432 |
| Peripheral Vascular Disease | 386 | 6.6% | 156 | 8.4% | 60 | 5.2% | 0.001 |
| Psychoses | 154 | 2.6% | 25 | 1.4% | 24 | 2.0% | 0.005 |
| Pulmonary Hypertension | 29 | 0.5% | 14 | 0.8% | 8 | 0.7% | 0.365 |
| Renal Failure | 677 | 11.6% | 268 | 14.5% | 179 | 15.4% | <0.001 |
| Rheumatic Disease | 165 | 2.8% | 136 | 7.4% | 84 | 7.3% | <0.001 |
| Solid Tumor | 64 | 1.1% | 20 | 1.1% | 31 | 2.6% | <0.001 |
| Valvular Disease | 406 | 6.9% | 129 | 7.0% | 94 | 8.1% | 0.365 |
| Venous Thromboembolism | 205 | 3.5% | 85 | 4.6% | 70 | 6.0% | <0.001 |
| Weight Loss | 403 | 6.9% | 107 | 5.8% | 76 | 6.5% | 0.268 |

ORIF: open reduction internal fixation. THA: total hip arthroplasty. HIV/AIDS: human immunodeficiency virus/acquired autoimmune disease syndrome.

Supplementary Table S4. Elixhauser comorbidity index data (ages 45-64)

|  | **ORIF**  **N=12,523** | | **THA**  **N=906** | | **ORIF+THA**  **N=435** | | **P-value** |
| --- | --- | --- | --- | --- | --- | --- | --- |
|  | **N** | **%** | **N** | **%** | **N** | **%** |  |
| Alcohol Abuse | 1,761 | 14.0% | 64 | 7.0% | 45 | 10.4% | <0.001 |
| Arrhythmia | 138 | 1.1% | 51 | 5.6% | 10 | 2.3% | <0.001 |
| Blood Loss Anemia | 145 | 1.1% | 20 | 2.2% | 25 | 5.7% | <0.001 |
| Cerebrovascular Accident | 281 | 2.2% | 25 | 2.8% | 25 | 5.7% | <0.001 |
| Chronic Peptic Ulcer Disease | 10 | 0.1% | 5 | 0.6% | 5 | 1.1% | <0.001 |
| Chronic Pulmonary Disease | 868 | 6.9% | 70 | 7.7% | 35 | 8.0% | <0.001 |
| Coagulopathy | 630 | 5.0% | 39 | 4.3% | 4 | 1.0% | <0.001 |
| Congestive Heart Failure | 366 | 2.9% | 31 | 3.4% | 20 | 4.6% | 0.0996 |
| Deficiency Anemia | 715 | 5.7% | 98 | 10.8% | 45 | 10.3% | <0.001 |
| Depression | 1,320 | 10.5% | 138 | 15.2% | 49 | 11.3% | <0.001 |
| Diabetes, complicated | 789 | 6.3% | 35 | 3.9% | 45 | 10.3% | <0.001 |
| Diabetes, uncomplicated | 1,576 | 12.5% | 149 | 16.4% | 55 | 12.6% | 0.004 |
| Drug Abuse | 1,009 | 8.0% | 39 | 4.4% | 25 | 5.7% | 0.003 |
| Fluid and Electrolyte Disorders | 2,667 | 21.2% | 177 | 19.5% | 120 | 27.6% | 0.003 |
| Hemiplegia/Paraplegia | 84 | 0.7% | 5 | 0.6% | 5 | 1.1% | 0.436 |
| HIV/AIDS | 30 | 0.2% | 0 | 0.0% | 0 | 0.0% | N/A |
| Hypertension, complicated | 442 | 3.5% | 60 | 6.6% | 20 | 4.6% | <0.001 |
| Hypertension, uncomplicated | 4,693 | 37.3% | 465 | 51.3% | 216 | 49.6% | <0.001 |
| Hypothyroidism | 692 | 5.5% | 89 | 9.8% | 35 | 8.0% | <0.001 |
| Liver Disease | 368 | 2.9% | 29 | 3.2% | 5 | 1.1% |  |
| Lymphoma | 10 | 0.1% | 5 | 0.6% | 1 | 0.2% | <0.001 |
| Metastatic Cancer | 40 | 0.3% | 15 | 1.7% | 15 | 3.5% | <0.001 |
| Myocardial Infarction | 414 | 3.3% | 20 | 2.2% | 25 | 5.7% | 0.003 |
| Obesity | 1,813 | 14.4% | 239 | 26.3% | 100 | 23.0% | <0.001 |
| Osteoporosis | 321 | 2.6% | 53 | 5.9% | 35 | 8.1% | <0.001 |
| Other neurological disorders | 661 | 5.2% | 90 | 9.9% | 25 | 5.8% | <0.001 |
| Peripheral Vascular Disease | 375 | 3.0% | 15 | 1.7% | 5 | 1.1% | 0.006 |
| Psychoses | 442 | 3.5% | 20 | 2.2% | 25 | 5.8% | 0.004 |
| Pulmonary Hypertension | 54 | 0.4% | 9 | 0.9% | 5 | 1.1% | 0.008 |
| Renal Failure | 351 | 2.8% | 50 | 5.5% | 10 | 2.3% | <0.001 |
| Rheumatic Disease | 208 | 1.7% | 61 | 6.7% | 20 | 4.6% | <0.001 |
| Solid Tumor | 40 | 0.3% | 5 | 0.6% | 5 | 1.1% | 0.011 |
| Valvular Disease | 208 | 1.6% | 25 | 2.8% | 15 | 3.4% | 0.002 |
| Venous Thromboembolism | 140 | 1.1% | 45 | 5.0% | 15 | 3.4% | <0.001 |
| Weight Loss | 435 | 3.5% | 35 | 3.9% | 25 | 5.7% | 0.0378 |

ORIF: open reduction internal fixation. THA: total hip arthroplasty. HIV/AIDS: human immunodeficiency virus/acquired autoimmune disease syndrome.
