# Supplementary material for: Rising incidence of acute total hip arthroplasty for primary and adjunctive treatment of acetabular fracture in older and middle-aged adults
Source: Eur J Orthop Surg Traumatol. 2023 Jul 22;34(7):3509–21. doi: 10.1007/s00590-023-03653-4 (PMC11490425; doi:10.1007/s00590-023-03653-4)
Supplement: Supplementary file 2 — STROBE diagram of cohort selection. Given non-specificity of ICD-9-PCS coding for acetabular fractures, patients with concomitant fractures* (clavicular, patellar, pelvic, proximal femoral, sacral, and scapular) were excluded from analysis. (DOCX 25 kb) [file 590_2023_3653_MOESM2_ESM.docx]

**Supplementary Fig. 1**

Concomitant fractures*, n=27,028

Age <45, n= 9,696

Non-operative treatment/>3 weeks until operative treatment, n= 27,214

Nationwide Inpatient Sample 2010-2020

Age ≥65

ORIF n= 1,171

THA n= 370

ORIF+THA n= 233

Age 45-64

ORIF n= 2,524

THA n= 182

ORIF+THA n= 87

Acetabular fracture

n= 68,507

Operatively treated acetabular fractures in patients ≥45 years old

n= 4,569
