# Supplementary material for: Rising incidence of acute total hip arthroplasty for primary and adjunctive treatment of acetabular fracture in older and middle-aged adults
Source: Eur J Orthop Surg Traumatol. 2023 Jul 22;34(7):3509–21. doi: 10.1007/s00590-023-03653-4 (PMC11490425; doi:10.1007/s00590-023-03653-4)
Supplement: Supplementary file 3 — Acetabular treatment trends by open reduction internal fixation (black), total hip arthroplasty (dark grey), and open reduction internal fixation + total hip arthroplasty (light grey) in the NIS database from 2010 to 2020 by admitting hospital type. a. rural hospital admissions in ages ≥65 b. urban non-teaching hospital admissions in ages ≥65 c. urban teaching hospital admissions in ages ≥65 d. rural hospital admissions in ages 45–64 e. urban non-teaching hospital admissions in ages 45–64 f. urban teaching hospital admissions in ages 45–64 (DOCX 29 kb) [file 590_2023_3653_MOESM3_ESM.docx]

**Supplementary Fig. 2**

a.

b.

c.

d.

e.

f.
